# Targeting p53–MDM2 interaction by small-molecule inhibitors: learning from MDM2 inhibitors in clinical trials

**DOI:** 10.1186/s13045-022-01314-3

**Published:** 2022-07-13

**Authors:** Haohao Zhu, Hui Gao, Yingying Ji, Qin Zhou, Zhiqiang Du, Lin Tian, Ying Jiang, Kun Yao, Zhenhe Zhou

**Affiliations:** 1 The Affiliated Wuxi Mental Health Center of Jiangnan University, Wuxi Tongren International Rehabilitation Hospital, Wuxi, 214151 Jiangsu China; 2 Jiangyin People’s Hospital, Wuxi, 214400 Jiangsu China

**Keywords:** p53, MDM2, Inhibitor, Multi-target, Degrader

## Abstract

p53, encoded by the tumor suppressor gene TP53, is one of the most important tumor suppressor factors in vivo and can be negatively regulated by MDM2 through p53–MDM2 negative feedback loop. Abnormal p53 can be observed in almost all tumors, mainly including p53 mutation and functional inactivation. Blocking MDM2 to restore p53 function is a hotspot in the development of anticancer candidates. Till now, nine MDM2 inhibitors with different structural types have entered clinical trials. However, no MDM2 inhibitor has been approved for clinical application. This review focused on the discovery, structural modification, preclinical and clinical research of the above compounds from the perspective of medicinal chemistry. Based on this, the possible defects in MDM2 inhibitors in clinical development were analyzed to suggest that the multitarget strategy or targeted degradation strategy based on MDM2 has the potential to reduce the dose-dependent hematological toxicity of MDM2 inhibitors and improve their anti-tumor activity, providing certain guidance for the development of agents targeting the p53–MDM2 interaction.

## Introduction

Protein–protein interactions (PPIs) play an important role in almost all biological activities (such as DNA synthesis and cell signal transduction), which can promote or inhibit the occurrence, development and metastasis of tumors. Therefore, intervention of PPI is a potential strategy in the field of tumor therapy [1]. However, since PPI is an interaction between two proteins, the binding surfaces of PPI are usually different from those of common small-molecule targets. The characteristics of large binding surfaces and dispersed activity sites make the development of small-molecule inhibitors extremely difficult for PPIs, which have long been known as ‘undruggable targets’ [2]. In recent years, studies have found that most PPIs have ‘hot spots’ [3]. According to the strategy of structure-based design or virtual screening in their hot spots, small-molecule inhibitors targeting PPIs, such as VHL (von Hippel–Lindau)-HIF1α (hypoxia inducible factor-1α) and Keap1 (Kelch-like ECH-associated protein 1)-Nrf2 (nuclear factor E2-related factor 2) [4, 5], have been successfully developed and have ideal clinical application prospects.

**Fig. 1:**
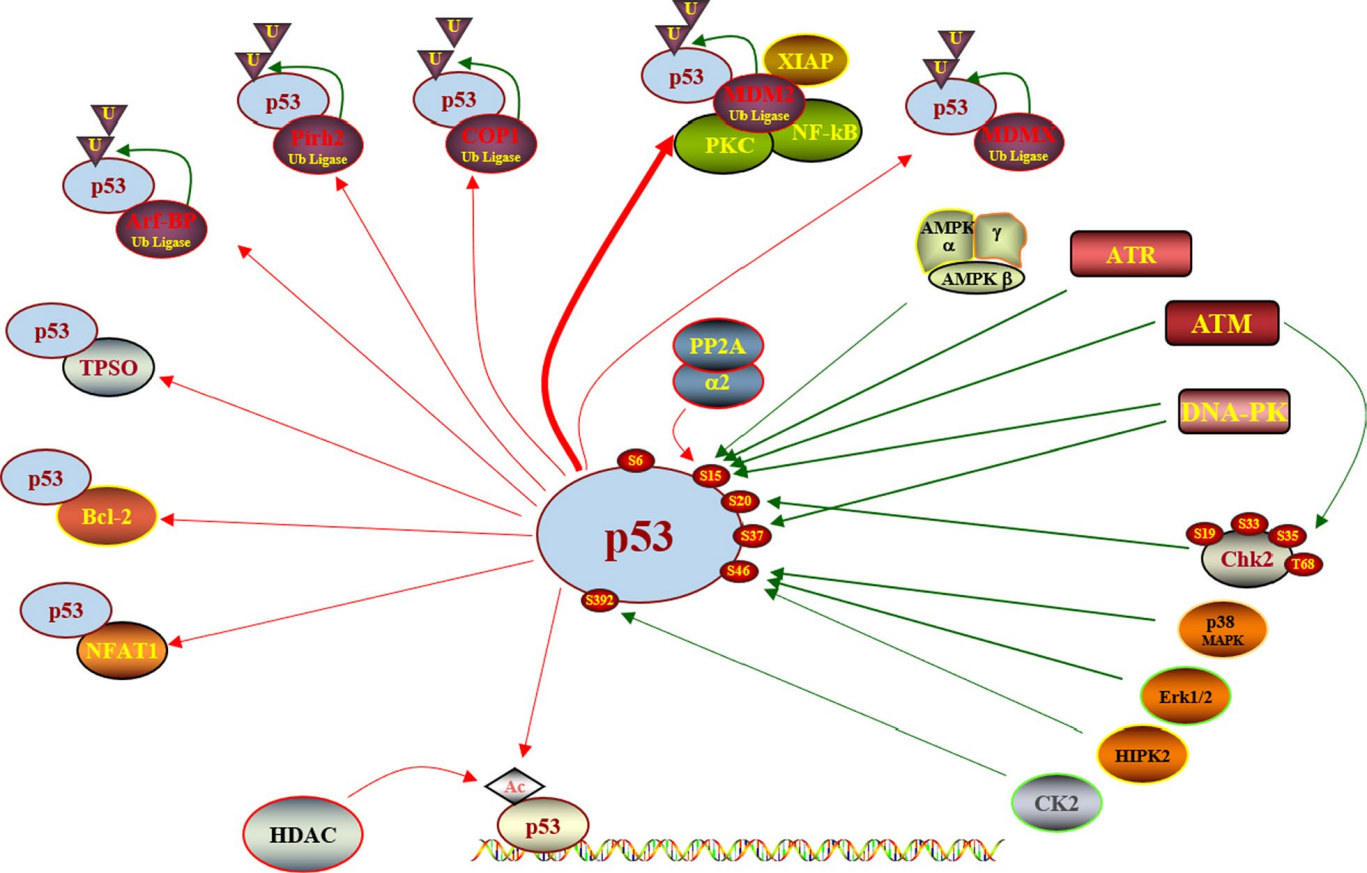
Proteins related to the regulation of p53 function

The p53–MDM2 (mouse double minute 2) protein interaction is an important target in the development of anti-tumor drugs. As one of the most important tumor suppressors, the p53 protein is inactivated or mutated in more than 50% of cancer cells. It plays an important role in the regulation of tumor cell cycle, apoptosis and DNA repair directly or induces the expression of downstream targets. The activation of the p53-dependent pathway caused by internal or external cell stress signals affects the occurrence, development and metastasis of cancer cells and prevents the proliferation of damaged cells with carcinogenic potential. In addition, as a transcription factor, a variety of genes can be activated by p53 to promote these tumor-related specific processes. Several proteins are closely related to the regulation of the function of p53, such as MDM2, MDMX, TPSO (translocator protein), Bcl-2 (B-cell lymphoma-2), and NFAT1 (nuclear factor of activated T-cells 1). (Fig. 1) [6]. Studies have revealed that after inactivation of the TP53 gene (encoding p53) in mice, cells lacking functional p53 cannot respond appropriately to external stimuli, resulting in a high probability of tumors [7]. Therefore, the targets related to the regulation of p53 protein expression or activation are of great research value. The MDM2 protein is widely studied because it can directly regulate the expression of p53 protein through negative feedback.

**Fig. 2:**
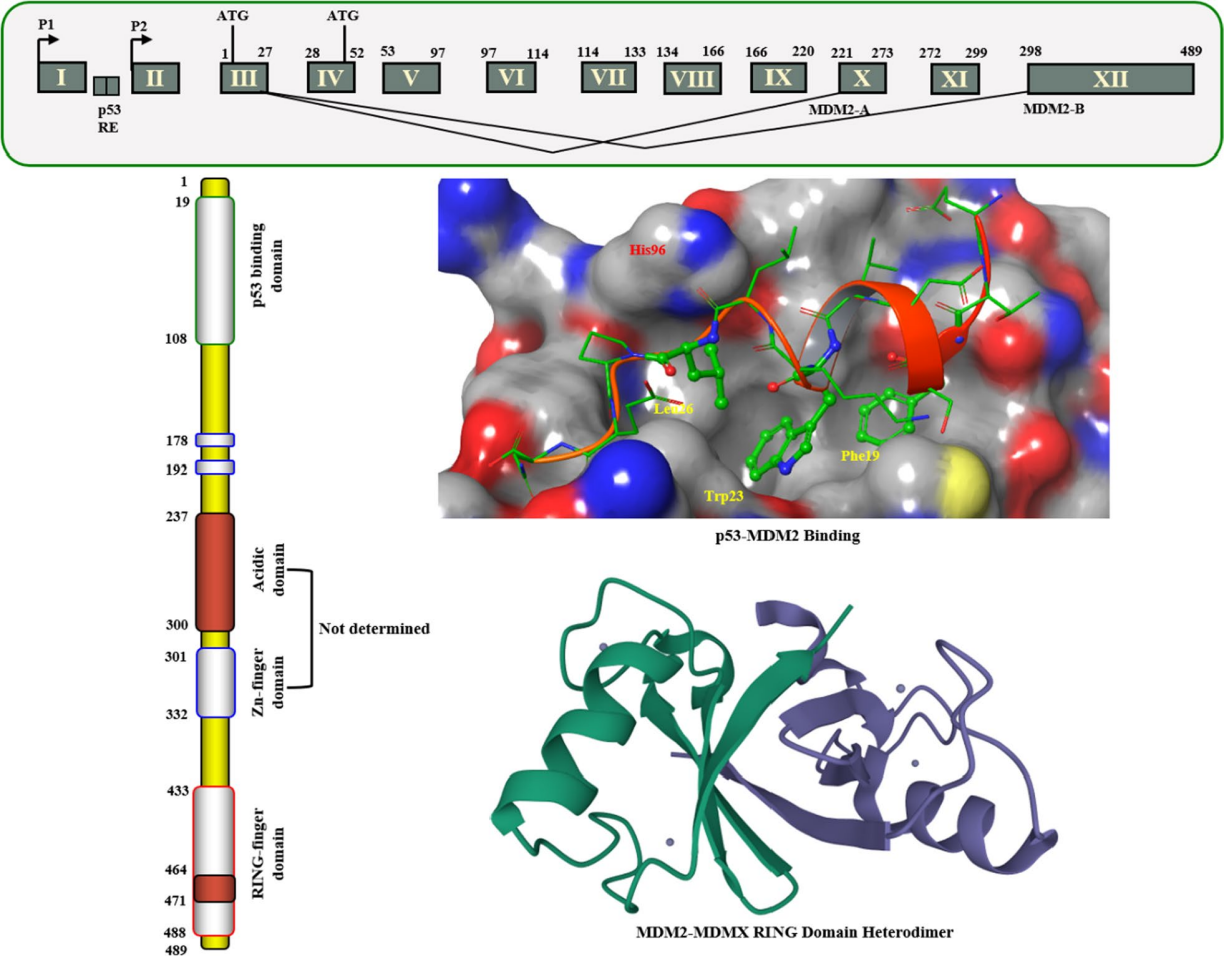
Structure of MDM2 gene and protein

The MDM2 gene locates on the long arm 13 ~ 14 of chromosome 12 (12q13 ~ 14), with a full length of 2372 kb, including 12 exons, encoding a protein containing 498 amino acids (Fig. 2). The MDM2 protein has four functional regions: region I includes approximately 100 amino acid residues at the N-terminus, and in addition to binding to the p53 protein, it can also directly bind to gene promoters to activate gene transcription; region II is a highly acidic region that can bind to ribosomal 15 protein and 5 s rRNA; region III contains a zinc finger structure, which has the activity of transcription factors and promotes cells from G1 phase to S phase; and region IV contains a RING finger structure that can mediate the interaction with p53 and bind to DNA or RNA to participate in cell cycle regulation and promote cell proliferation [8, 9]. The ATM-dependent phosphorylation of MDM2 allows the overexpressed MDM2 to bind p53 mRNA and promote p53 translation [10]. The negative feedback regulation of MDM2 protein has many mechanisms. First, the MDM2 protein can bind to the p53 protein through PPI to prevent its expression under stress conditions and then inactivate or weaken its transcriptional function. Second, the MDM2 protein has a unique RING domain, which can promote the transfer of p53 protein from the nucleus to the cytoplasm and reduce its accumulation in the nucleus, leading to the disappearance of p53 protein transcription function. Finally, although p53 is mainly polyubiquitinated by UBE4B (ubiquitination factor E4B, an E4 ligase) for proteasomal degradation, MDM2 can cooperate with MDMX (mouse double minute X) to kinetically enhance p53 polyubiquitination, resulting in p53 stability and the occurrence of tumors [11–13]. Therefore, regulation of the p53–MDM2 protein interaction can effectively regulate the expression and function of the p53 protein.

**Fig. 3:**
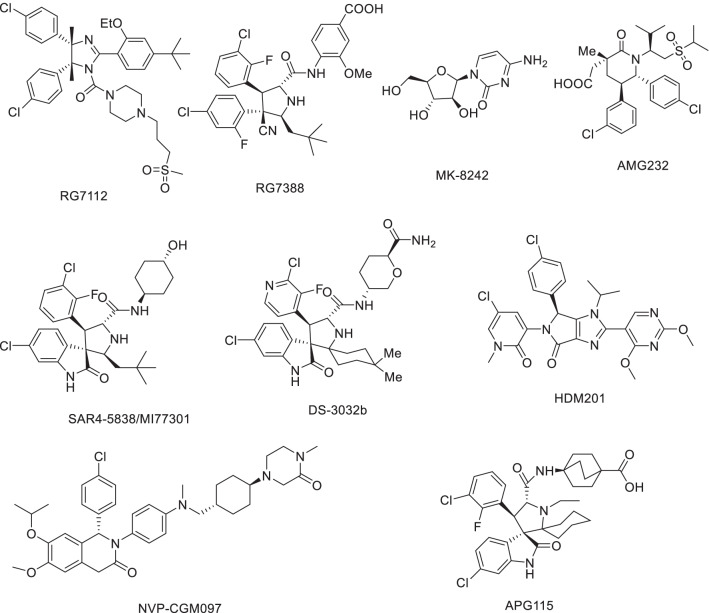
Nine MDM2 inhibitors in clinical trials

The cocrystal structure of the N-terminal transcriptional activation region of MDM2 and p53 protein showed (PDB: 1YCR, Fig. 2) that the binding surface area of p53 and MDM2 protein was small, and there was an obvious binding cavity. Further verification by alanine mutation scanning has shown that the residues of three amino acids of p53, Phe19, Trp23 and Leu26 were deeply inserted into the binding pocket of p53 and MDM2, which were, respectively, so that they were closely bound by physical action [14]. According to these three clear binding sites, a variety of structural types of MDM2 inhibitors have been reported, among which nine have entered clinical trials (RG7112, RG7388/RO6839921, MK-8242, AMG232, SAR405838, DS-3032b, HDM201, NVP-CGM097 and APG-115) (Fig. 3), with a variety of scaffold types [15–18]. For six of these, the details of their discovery and the structural modification of the lead compounds have been reported. (RO6839921 is the inactive prodrug of RG7388.) The discovery process of HDM201 has been generally reported, while MK-8242 and DS-3032b have no specific literature to report the source of this molecule. Therefore, this review mainly focuses on the seven compounds with detailed research and development processes from the perspective of medicinal chemistry and summarizes the discovery of the lead compounds, structural optimization, and preclinical and clinical results.

## RG7112

**Fig. 4:**
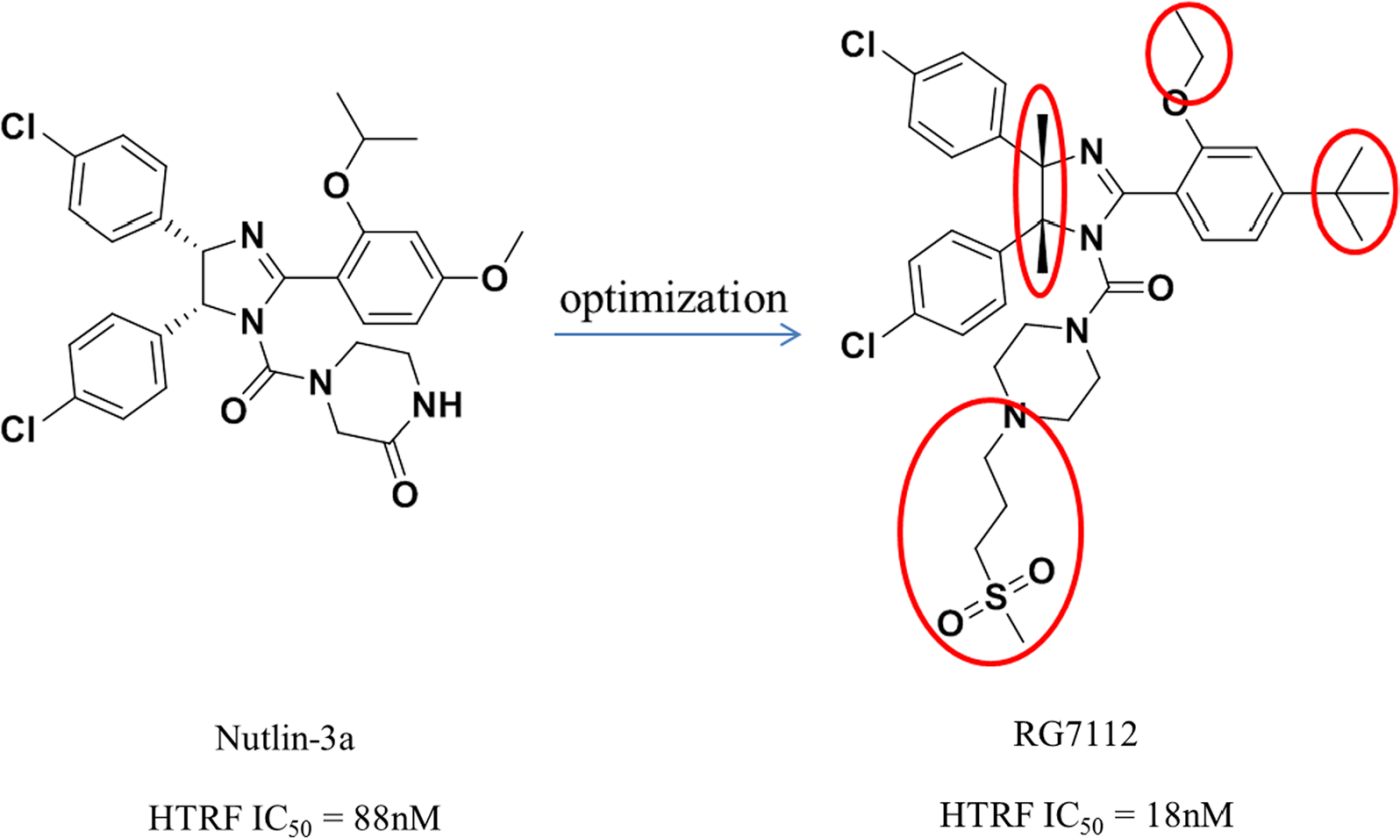
Discovery of RG7112

RG7112 was modified by Roche based on the structure of Nutlins and was the first to be introduced into clinical trials (Fig. 4) [19]. Nutlins have an imidazoline scaffold, which was reported to have been obtained through high-throughput screening in 2004 [20]. Nutlin-3a had the strongest activity, and its binding activity with MDM2 reached 90 nM. At the same time, the crystal structure of the complex of the first small molecule with MDM2 protein (PDB ID: 1RV1) was analyzed, and the binding mode of MDM2 with small-molecule inhibitors was revealed for the first time, which laid the foundation for the subsequent discovery of MDM2 inhibitors. Superimposition of Nutlin-2 on the crystal structure of the p53–MDM2 has shown that the main active sites of Nutlin-2 binding to MDM2 are consistent with the results of alanine mutation scanning experiments, which have shown that p53 primarily occupies the binding cavity in MDM2 through residues Phe19, Trp23 and Leu26, indicating that small-molecular compounds (such as Nutlin) can simulate the α-helix of p53 protein in solution or in vivo to occupy the key binding cavity of p53 and MDM2 protein, thereby inhibiting the activity of MDM2 protein and releasing p53 protein. Nutlin-3a can compete for binding sites in the N-terminus of MDM2 protein and p53 (Phe19, Trp23, Leu26) to block the binding between MDM2 and p53 and reduce the degradation of p53, which then results in induction of apoptosis of tumor cells, reversal of the immunosuppressive microenvironment and triggering of immunogenic cell death [21].

Nutlin-3a can activate wild-type p53 protein in tumor and normal cells at the same time and selectively inhibit tumor cells. Its antiproliferative activity against a variety of wild-type p53 inactivated tumor cells is 1–2 μM (including HCT116, RKO and SJSA-1), but its activity against mutant p53 tumor cells (MDA-MB-435 and SW480) is not obvious [22]. It has strong selectivity, which is similar to the results of the polypeptide inhibitors of MDM2 protein, which is wild-type p53 dependent. The in vivo efficacy showed that Nutlin-3a can activate p53 protein in a dose-dependent manner and induce the expression of p53-related genes (p21 and MDM2). At 200 mg/kg, twice a day, 20 days after oral administration of Nutlin-3a, the tumor growth inhibition rate reached 90%, with no obvious side effects [20].

**Fig. 5:**
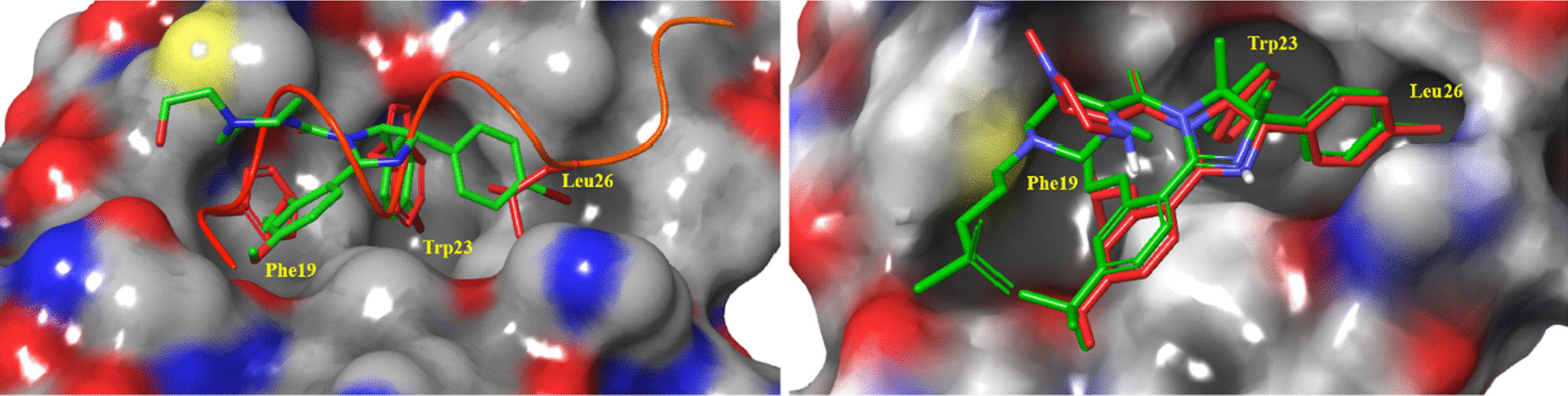
**A** Comparison of binding patterns between Nutlin-2/p53 and MDM2 protein (PDB ID: 1RV1/1YCR); **B** comparison of protein binding patterns between Nutlin-3a/RG7112 and MDM2 (PDB ID: 4J3E/4IPF)

Further structural optimization was carried out to maintain the most important structural characteristics of its binding to the MDM2 protein and investigate the effect of other side chain groups on its activity. The crystal structure of the complex of Nutlin-3a and MDM2 protein showed that its p-chlorophenyl was perfectly embedded in the active pockets of Leu26 and Trp23 and that isopropyloxy occupied the cavity of Phe19, demonstrating retention of the three key groups. To prevent the imidazoline ring from being oxidized to imidazoline ring, a methyl was added at its 4 and 5 positions. In vitro metabolism studies showed that the methoxy group of Nutlin-3a was unstable and was therefore substituted with tert-butyl. At the same time, to reduce the molecular weight of the target compound, the ethoxy was replaced with isopropyloxy, based on the crystal structure of Nutlin-2 and MDM2; after the above groups were replaced, the effects of exposure to solvent area were investigated, and the effects of different polar groups on their binding to MDM2 and the pharmacokinetic parameters were determined. Finally, through the activity and pharmacokinetic evaluations, it was determined that RG7112 had a stronger binding ability to MDM2 than Nutlin-3a with an enhanced cell activity and better pharmacokinetic parameters, making it more suitable for clinical trials. The crystal complex structure of RG7112 and Nutlin-3a with MDM2 protein is shown in Fig. 5. The binding mode in the three key cavities is nearly the same, which verifies the stability of the MDM2 protein active pocket.

RG7112 has been demonstrated to have growth inhibition and killing effects on SJSA-1 osteosarcoma cells with a high expression of MDM2 protein in vitro. Dose-dependent cell cycle arrest was induced in HCT116 and SJSA1 cells at the G1 and G2/M phases [23]. In vivo experiments showed that RG7112 (25 ~ 200 mg/kg, oral administration) activated the p53 pathway in vivo and induced apoptosis of tumor cells. RG7112 (100 mg/kg^−1^, once a day, 5 days/week, for 3 weeks) reduced the tumor growth rate and improve the survival rate in the GBM model [24]. At present, RG7112 has been tested in a variety of clinical trials, mainly including chronic myeloid leukemia (CML), acute myeloid leukemia (AML), solid tumors and hematological tumors. Although the levels of p53 and downstream p21 in patients with liposarcoma treated with this drug were significantly increased, one case of PR and 14 cases of SD were found in 17 assessable patients. However, at least one adverse reaction occurred in all patients, including 12 serious adverse reactions, including neutropenia and thrombocytopenia in eight patients [25]. Another phase I clinical trial for leukemia patients also proved that RG7112 treatment can improve the expression level of p53 and downstream genes. In 30 patients, five cases of CR or PR and nine cases of SD were observed [26]. However, the follow-up test of RG7112 was not carried out due to the poor tolerability and relatively severe hematologic and gastrointestinal toxicities at the required high doses.

## RG7388

**Fig. 6:**
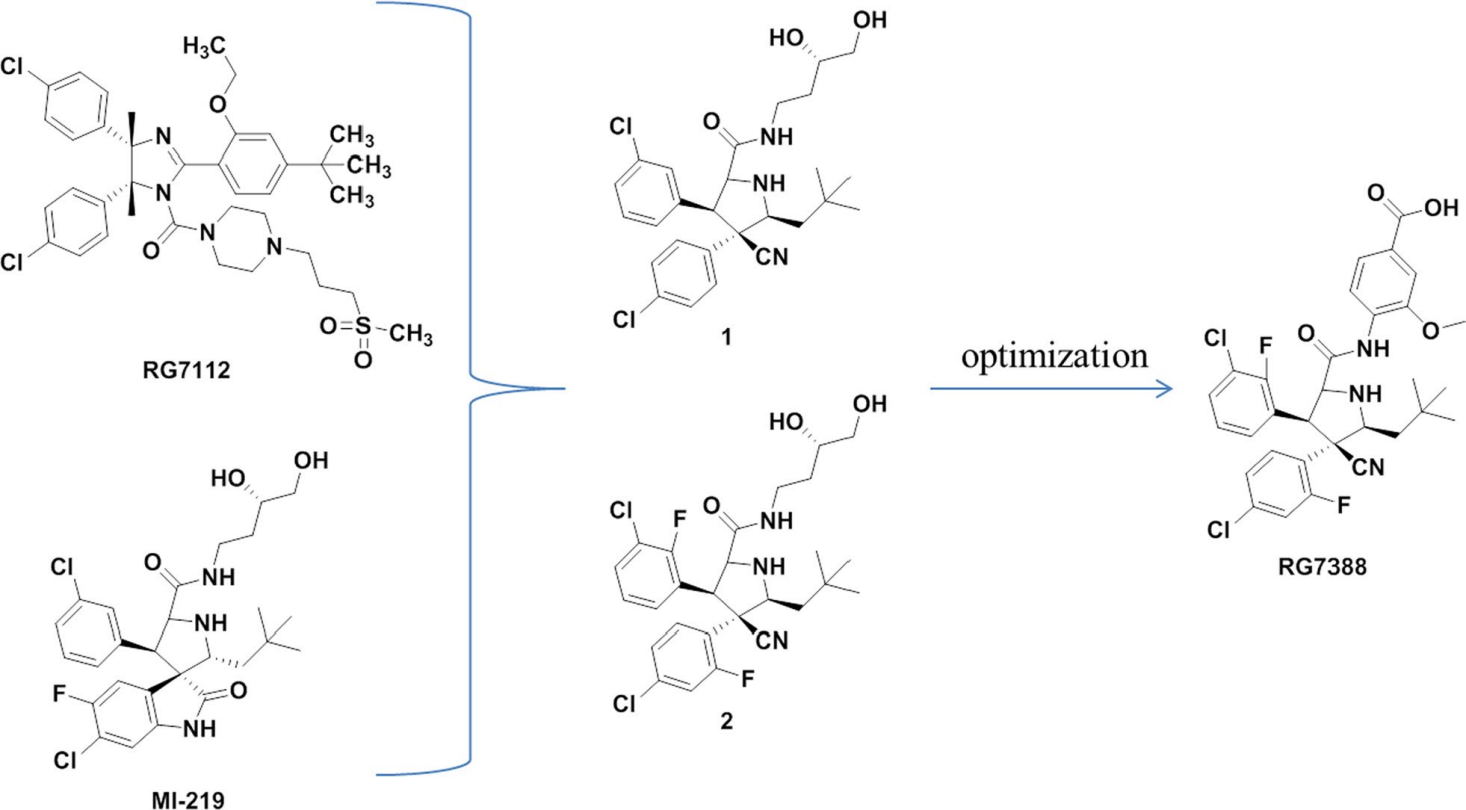
Discovery of RG7388

Roche’s researchers observed that the two benzene rings bound to the active cavities of Trp23 and Leu26 in the structure of the reported MDM2 inhibitors were all cis-conformations, while MDM2 inhibitors with trans-conformations were not reported. Therefore, based on MI-219 [27, 28] and RG7112, these researchers designed and synthesized Compound 1 with trans-conformations of the two key benzene rings Fig. 6), in which cyano was crucial to maintain its conformation and activity. After structural optimization, the second MDM2 inhibitor (RG7388) to enter clinical trials was obtained [29].

**Fig. 7:**
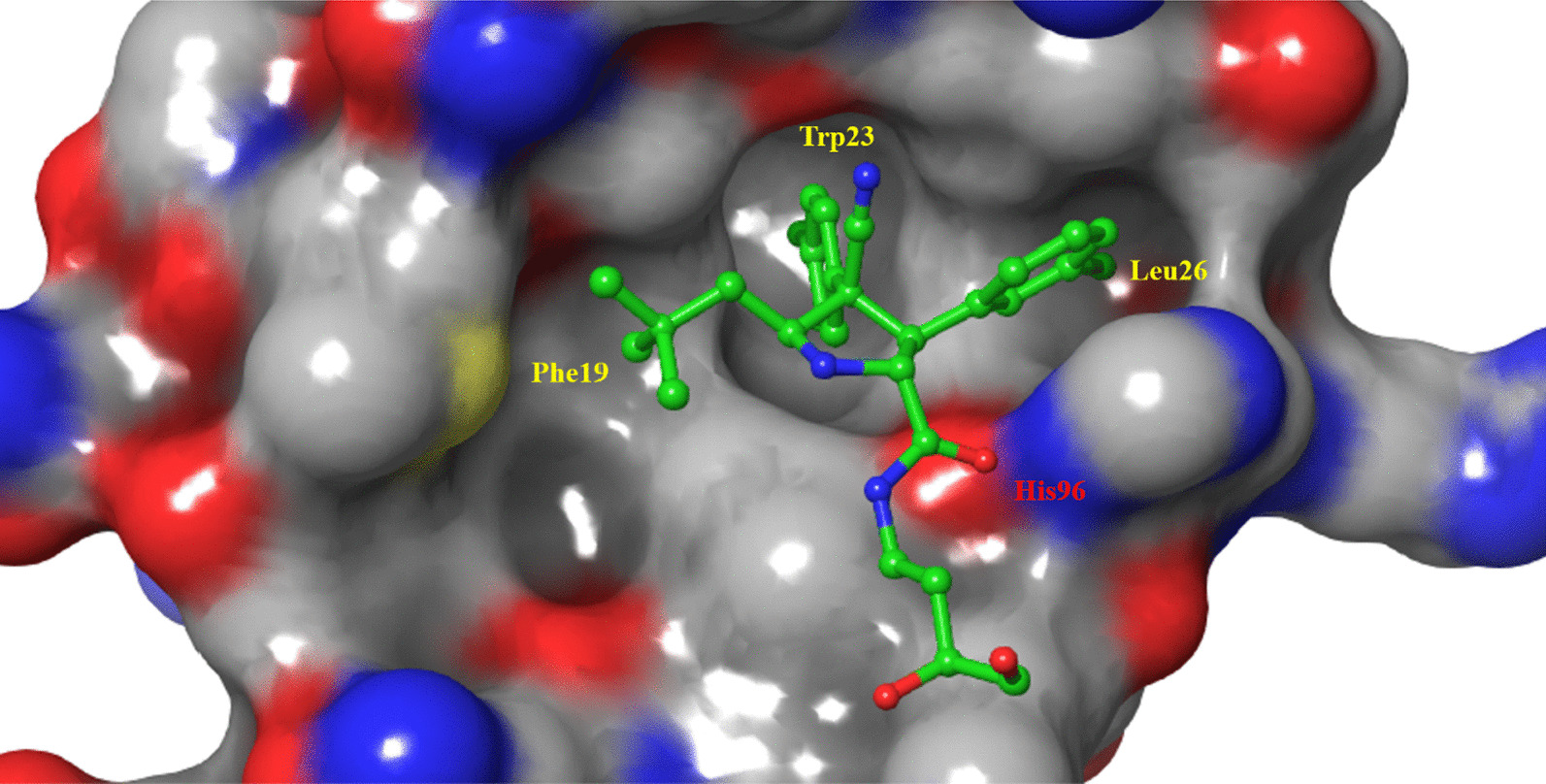
The binding mode of Compound 1 with MDM2 (PDB ID: 4JRG)

The cocrystal structure of Compound 1 and MDM2 protein showed (PDB ID: 4JRG) (Fig. 7) that Compound 1 occupied the three key active cavities of MDM2 protein, and its binding activity with MDM2 protein was 196 nM (IC_50_, HTRF). Compound 1 showed moderate inhibitory activity against wild-type p53 tumor cells, but no obvious inhibitory activity against p53 mutant tumor cells. After a systematic structure–activity relationship study, Compound 2 showed higher selectivity and activity than Compound 1. However, due to the high clearance rates and low oral bioavailability of Compounds 1 and 2, the structure of the groups exposed to the solvent region was optimized to improve its PK parameters. Finally, the second MDM2 inhibitor, RG7388, was obtained, which had higher binding ability, cell activity, microsome stability and PK parameters. The binding activity of RG7388 to MDM2 reached 6 nM, and the antiproliferative activity against various tumor cells with high expression of wild-type p53 was approximately 300 nM. The selectivity over p53 mutant tumor cells was greater than 100 times. The oral bioavailability in mice reached 80%, half-life was 1.6 h, and the metabolic stability was better than those of Compounds 1 and 2.

RG7388 can effectively activate the p53 pathway, lead to wild-type p53 expression, activate cell cycle arrest or apoptosis, and inhibit tumor proliferation in nude mouse neuroblastoma xenograft experiments [30]. In the SJSA xenograft model of mice, RG7388 (25 mg/kg, oral administration) led to tumor growth inhibition and regression, and induced apoptosis and antiproliferative effects [31]. According to the China Pharmaceutical Pipeline Monitor database (CPM), RG7388 has been assessed in 15 clinical trials, of which the world 's highest state of research and development is phase III clinical trials (NCT02545283) in combination with cytarabine in the treatment of relapsed or refractory AML. The preliminary results showed that the complete remission rate reached 25%, and the median remission time was approximately 6.4 months, which was related to the level of MDM2 protein before treatment [32]. However, the study was discontinued due to unexpected results.

RO6839921 is an inactive PEGylated prodrug of RG7388, which was designed to improve the exposure variability and pharmacokinetic characteristics of RG7388. Intravenous injection of RO6839921 at a safe dose showed good anti-tumor activity in osteosarcoma and AML xenograft models [33]. Although phase I studies demonstrated improved pharmacokinetic parameters of RG7388 for advanced solid tumors and AML, its safety was comparable to that of RG7388 and did not show sufficient advantages [34, 35].

## SAR405838 (MI-77301) and APG-115

**Fig. 8:**
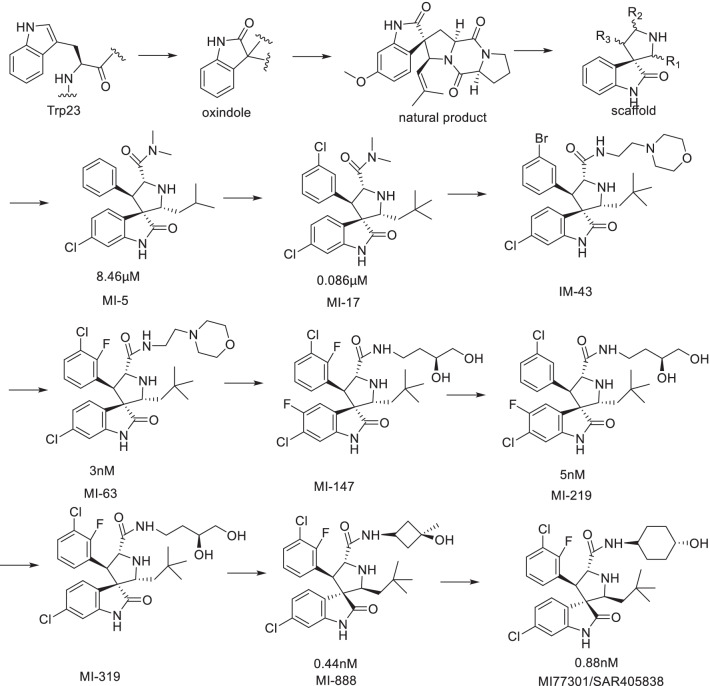
Discovery of MI77301

SAR405838 and APG-115 belong to spirooxindoles, which were developed by Wang Shaomeng's research group based on the protein binding of p53 and MDM2. The indole ring is the key to the binding between p53 and MDM2. The amino group in the indole ring can form hydrogen bonds with MDM2, and it was also found that the oxindole can perfectly simulate the binding mode of the Trp23 residue of p53 with MDM2 (Fig. 8). Taking into account the role of natural products in drug discovery, the team searched for the substructures of oxindole rings and found that many natural products have an oxindole ring (spirotryprostatin A and alstonisine). However, molecular docking showed that these natural products could not bind well to MDM2 protein, which keenly detected the possibility of spirooxindole rings as MDM2 inhibitors. As a rigid scaffold, spiropyrrolidine can be used as a carrier to simulate the three key amino acid residues of Phe19, Trp23 and Leu26. MI-5 was preliminarily obtained based on the structural design, and its three key residues that could simulate the binding of p53 to MDM2 were determined by molecular docking, but its binding ability was low (Ki = 8.46 μM). After replacing isobutyl for a 2,2-dimethylpropyl with stronger hydrophobic effect by structural optimization, compound MI-17, with a 98-fold activity enhancement, was obtained, which had strong selectivity and tumor inhibitory activity [27].

By comparing MI-17 with the most active peptide inhibitor at that time [36], it was found that the activity of MI-17 was nearly 100 times lower than that of the peptide inhibitor, indicating that there was still room for further optimization of MI-17. By analyzing the crystal complex structure of p53–MDM2, it was found that the Leu22 residue played a key role in the binding of p53 and MDM2 and that the Leu22 was located at a position not too deep into the binding pocket of MDM2. Therefore, based on MI-17, it can simulate the binding of Leu22 to MDM2 at the same time and increase polar groups to improve the physical and chemical properties of the target compounds. The binding activity of MI-63 with MDM2 was 3 nM, which was 2000 times higher than that of the p53 peptide (residues 13–29). MI-63 had high stereospecificity and selectively blocked the interaction of p53–MDM2 without affecting the interaction of Bcl-2-Bid. Its inhibitory effect on tumor was wild-type p53-dependent, and its toxicity to p53 knockout cells and normal cells was small. In addition, MI-63 can induce apoptosis of tumor cells in patients with chronic lymphocytic leukemia [37]. However, its PK constant is not suitable for in vivo studies. In order to improve the shortcomings of MI-63, the structure was optimized and MI-219 was obtained with the binding activity with MDM2 of 5 nM. The wild-type p53 protein was selectively activated in a variety of tumor cells, and the activity of mutant and knockout p53 tumor cells was not obvious. It had good oral bioavailability in both rats and mice and can activate p53 protein in mice. It can completely inhibit the growth of tumor cells in vivo, and however, it cannot cause tumor regression [28, 38].

Since the cell activity of MI-147 was higher than that of MI-219, structural optimization based on MI-147 was carried out to further improve its PK parameters and anti-tumor activity in vivo. Finally, MI-888 was obtained, which can completely and continuously eliminate the tumor. The trans conformation of the two benzene rings of MI-888 is the main reason for the enhanced binding ability of MI-888 to the MDM2 protein. Removing the fluorine substituent on the oxindole ring in MI-219 and retaining the fluorine substituent on the benzene ring can improve its PK parameters. At the same time, its polar tail extending into the solvent region can further improve its PK parameters in vivo and its binding ability to MDM2 protein (Ki = 0.44 nM). Its inhibitory activity against tumor cells was at the nM level in SJSA-1 and RS4;11 cells, and the selectivity for wild-type p53 over mutant or knockout p53 tumor cells was high, without significant toxic side effects [39].

**Fig. 9:**
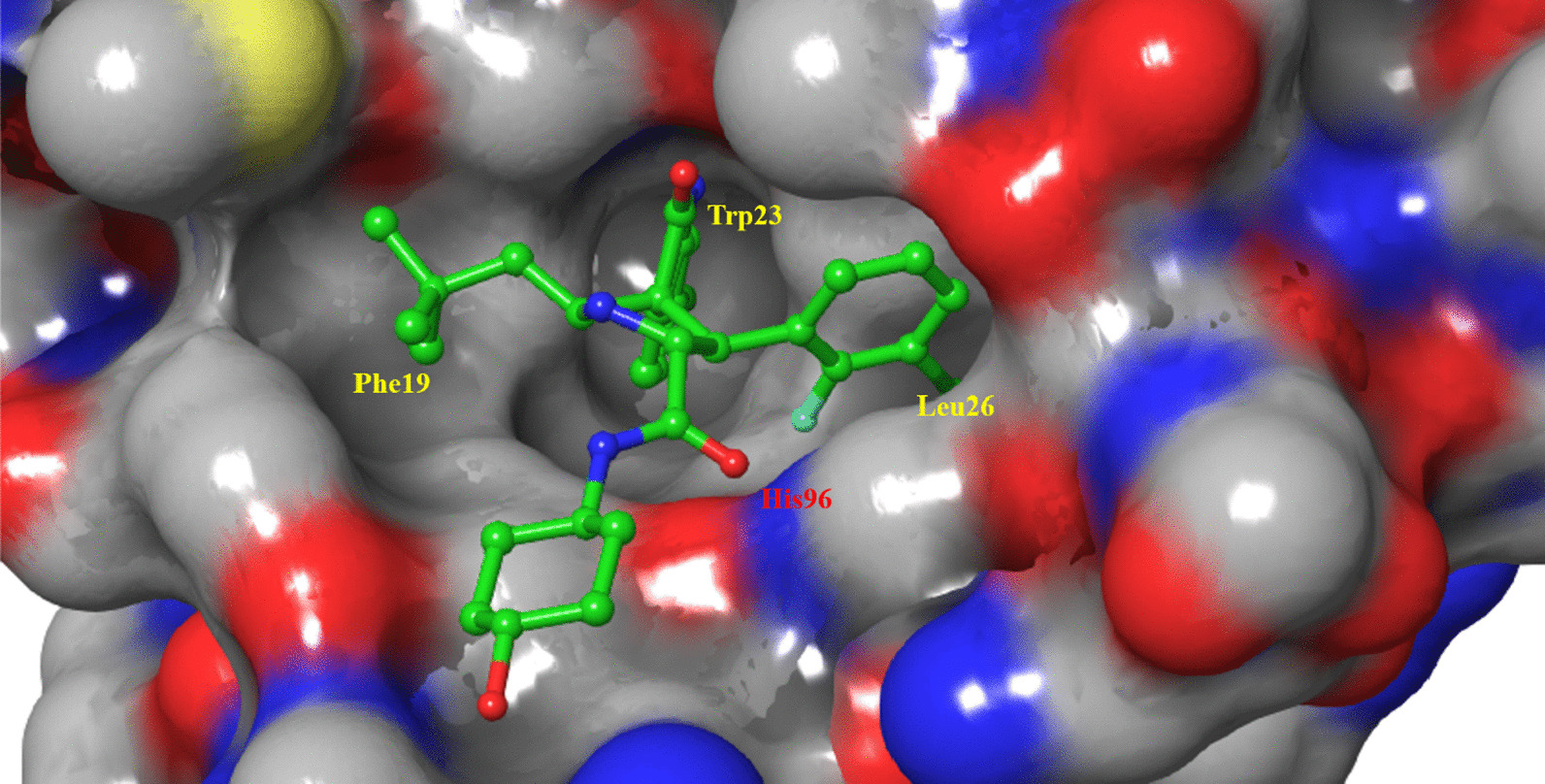
Binding pattern of MI77301 to MDM2 protein (PDB ID: 5TRF)

MI-77301 (SAR405838) was obtained by simply replacing the groups exposed to the solvent region of MI-888, and its binding activity with MDM2 reached 0.88 nM, with a high selectivity and high specificity over other proteins [40]. The cocrystal structure of SAR405838 and MDM2 protein showed (Fig. 9) that, in addition to simulating the three key amino acid residues of p53–MDM2, SAR405838 is also capable of other additional interactions and induces the refolding of the N-terminal region of unstructured MDM2 to achieve its high-affinity binding to MDM2. Wild-type p53 can be effectively activated in vitro and in xenograft tumor tissues of leukemia or solid tumor, leading to p53-dependent cell cycle arrest and apoptosis. In well-tolerated dose regimens, SAR405838 can lead to durable tumor regression by complete tumor growth inhibition in SJSA-1, RS4;11, LNCaP and HCT-116 xenograft models.

In the SJSA-1 model, SAR405838 was shown to effectively inhibit the growth of tumors with a high selectivity over p53 mutant or deleted cancer cell lines [40]. SAR405838 effectively inhibited cell growth and induced dose-dependent apoptosis in ABTR1 and ABTR2 sublines [41]. With a well-tolerated dose scheme, SAR405838 achieved persistent tumor regression or completely inhibited tumor growth in the xenograft models of SJSA1, RS411, LNCaP and HCT-116 mice [40]. Phase I clinical trials for patients with advanced solid tumors demonstrated that SAR405838 had good safety and pharmacokinetics, but was not able to be administered at the planned weekly maximum tolerance dose [42]. Further exploration of its potential in combination with the MEK1/2 (MAP kinase kinase 1/2) inhibitor pimasertib in the treatment of locally advanced or metastatic solid tumors did not find significant drug interactions [43]. However, its anti-tumor activity suggests inhibition of the MAPK (mitogen-activated protein kinase) pathway, while restoring p53 activity has potential significance in malignant tumors with wild-type TP53 and MAPK mutations.

The team further determined that the above spiroindole compounds could be transformed into four diastereoisomers in protonic solvents [44]. The possible mechanism was that the pyrrolidine ring could be opened by the anti-Mannich reaction, after which the above four diastereoisomers were formed by the Mannich reaction cyclization. Among them, IV was the most stable and most active diastereoisomer. However, the activity instability of the above MDM2 inhibitors was caused by the differential isomerization. Based on the above reasons, after the introduction of two identical substituents at C2, there were two kinds of diastereoisomers. Studies have shown that when the C2 was the introduced substituent, the compound was more likely to transform to the trans conformation (stable conformation). Finally, the structure was optimized to obtain MI-1061, and the binding activity of MI-1061 to MDM2 was 0.16 nM (Ki). It can effectively activate p53 protein and induce apoptosis in SJSA-1 xenograft tumor in mice, as well as tumor regression. As a result, a defect with high chemical stability in the solvent and breaking through the first generation of spirooxindole MDM2 inhibitors was obtained [45].

**Fig. 10:**
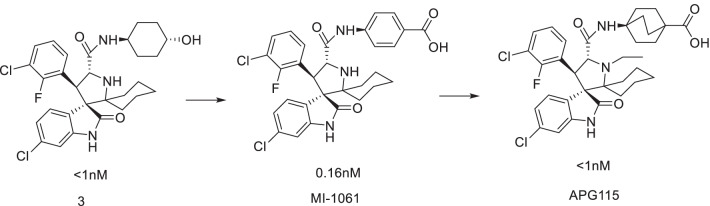
Discovery of APG-115

Compound 3 was used as the lead compound for a systematic structure–activity relationship study to obtain APG-115 (Fig. 10), which showed a strong binding ability with MDM2 protein (Ki < 1 nM) [46]. It could activate p53 protein in a variety of tumor cells and inhibit cell proliferation at nM concentrations, with an enhancement in its selectivity over p53 knockout tumor cells. APG-115 was very stable in solution and had optimal oral pharmacokinetic parameters. Single-dose oral administration of APG-115 could effectively activate the p53 protein in SJSA-1 xenograft tumor in mice and cause complete and permanent regression of the tumor, as well as in RS4;11 AML model.

APG-115 has obvious activity and drug administration advantages and overcomes the pharmaceutical defects of a slow isomerization reaction and poor solubility in a neutral environment in the early clinical compound SAR405838, with an activity greater than 10 times that of SAR405838. APG-115 affects progression by inducing G0/G1 arrest in AGS and MKN45 cells with wild-type p53, activating p53 to enhance the radiosensitivity of AGS and MKN45 cells, and inducing concentration-dependent G2/M arrest and S phase reduction in p53 wild-type cell lines (TPC-1 and KTC-1). In vivo experiments showed that APG-115 combined with radiotherapy could enhance the radioanti-tumor effect against gastric adenocarcinoma [47]. Phase I trials showed that APG-115 had good tolerance, controllable adverse reactions and good anti-tumor activity [48] and demonstrated the potential to mediate drug resistance in immunotherapy [49, 50]. A phase I trial in a Chinese population showed that APG-115 had good anti-tumor activity in the treatment of patients with MDM2 amplification and TP53-WT liposarcoma [51].

## AMG232

**Fig. 11:**
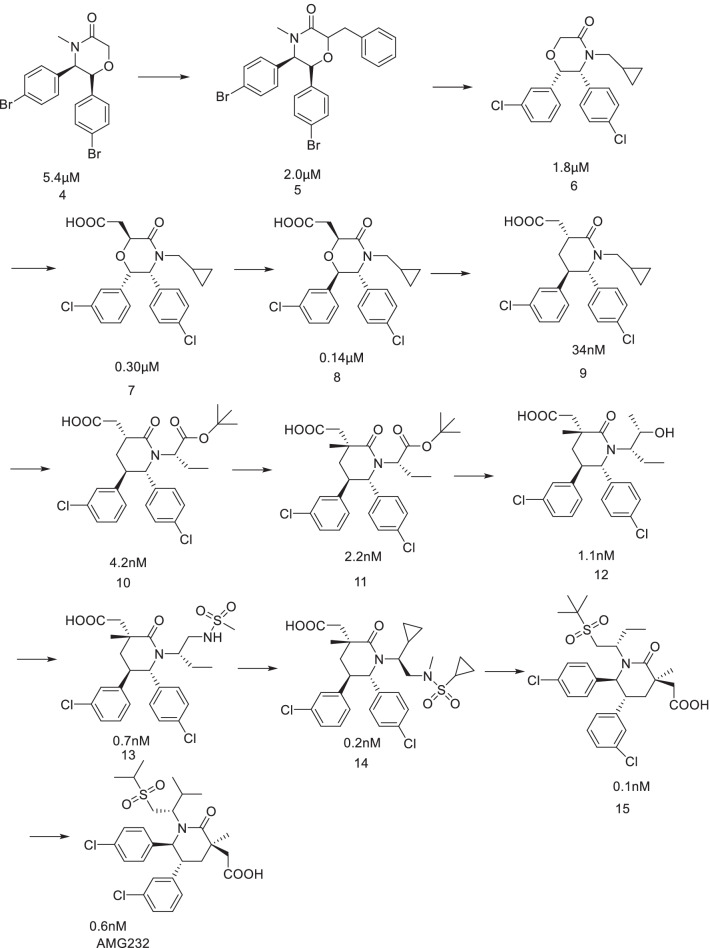
Discovery of AMG232

Researchers of Amgen designed and synthesized a series of six-membered ring scaffolds based on the structure of the existing MDM2 inhibitors (Fig. 11). After preliminary evaluation, it was determined that the morpholinone scaffold could well simulate the three key amino acid residues (IC_50_ = 5.4 μM) of p53 binding to MDM2, and the introduction of a benzyl group at the C2 position enhanced the activity of Compound 5. However, its cocrystal structure with MDM2 showed that it did not occupy the Leu26 cavity. Simulated spirooxindole compounds, the introduction of 3-chlorobenzene can better occupy the Leu26 cavity, and through the π–π bond with His96 binding, while the activity had a certain enhancement; further introduction of an acetic acid group at C2 indicated that it enhanced the binding ability of the compound to MDM2 through electrostatic interaction with the His96 residue [52].

**Fig. 12:**
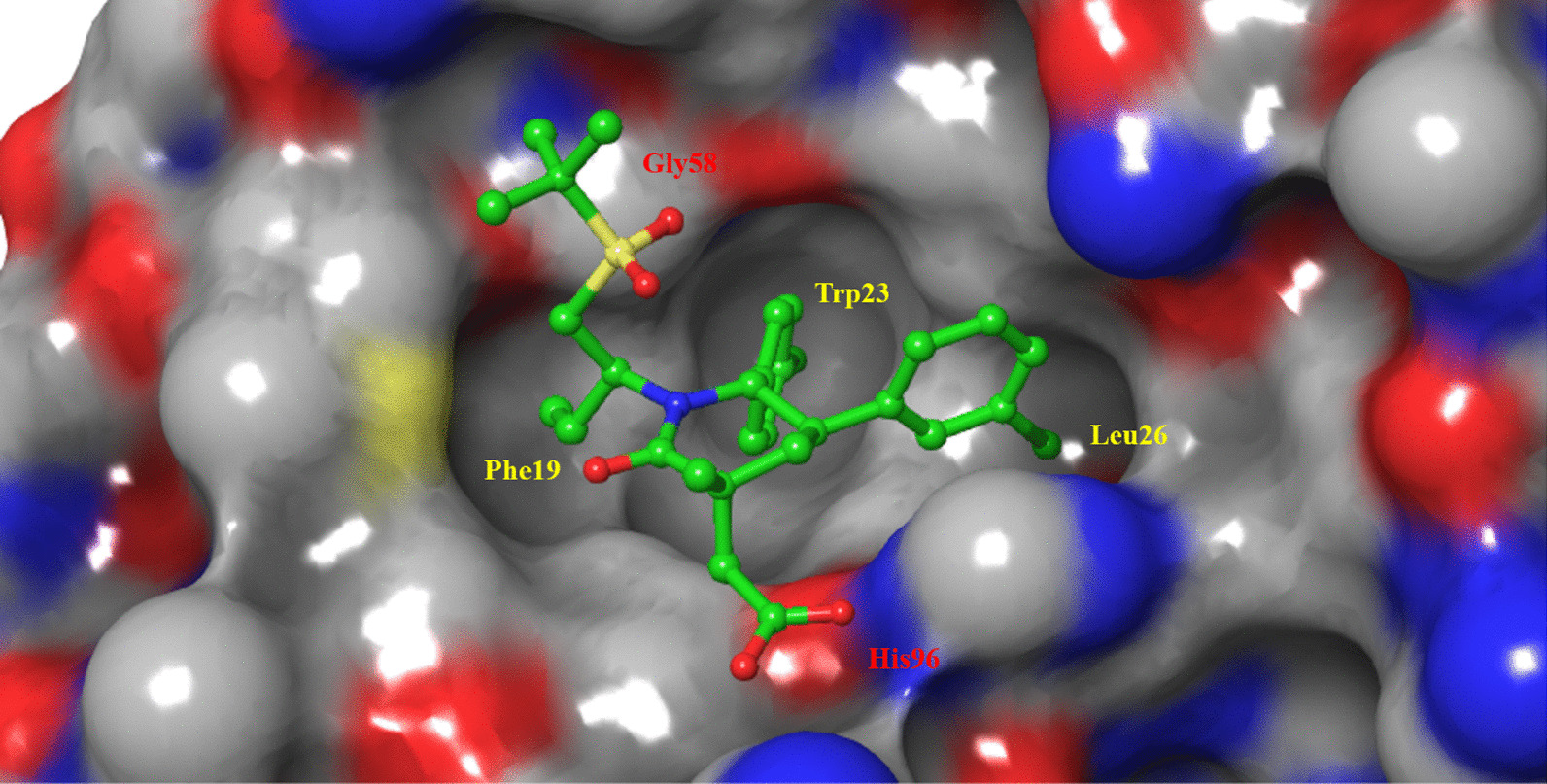
Binding pattern of Compound 12 with MDM2 protein (PDB ID: 4OAS)

Further studies showed that the stereo conformation of the compound was also crucial for its binding to MDM2. The morpholinone scaffold was replaced by a piperidone scaffold, and the conformation was fixed to obtain Compound 9. The cocrystallization of Compound 9 with MDM2 protein showed that cyclopropyl penetrated into the Phe19 cavity in the downwards direction. Therefore, the introduction of a Boc group at the position adjacent to cyclopropyl to fix the conformation of cyclopropyl significantly improved its IC_50_, and a methyl group at the 3 position was also introduced to stabilize the conformation. The structure–activity relationship of the lead compound was studied systematically. The substitution of the ester site for hydroxymethyl improved the activity to 1.1 nM, resulted in a low clearance rate (CL = 0.03 L/H/Kg) with higher target selectivity than Compound 11, and reduced the inhibition rates of CYP450, CYP3A4 and hPXR. Substituting its hydroxyl group with cyclopropylsulfonamide allowed binding of the compound to the Gly58 amino acid residue near the cavity of Phe19, further improving its affinity for MDM2 protein and its cellular activity. However, Compounds 13 and 14 had high clearance rates in rats and human hepatocytes. Then, the sulfonamide group was replaced by a more stable sulfone group, and its cocrystal structure with MDM2 protein was shown (Fig. 12). As expected, the compound could occupy the three key active cavities, occupy the Gly58 cavity and interact electrostatically effect with His96. Its metabolic stability was further improved, its intrinsic clearance rate was reduced, and its affinity for MDM2 protein and cell activity was also improved [53].

On this basis, simple structural optimization was carried out to obtain AMG232 [54], which had better PK parameters in rhesus monkeys, low inhibitory activities against CYP, CYP3A4 and PXR, low internal clearance rates in human hepatocytes and rats, and 2500 times higher selectivity for tumor cells with high expression of wild-type p53 than that for p53 knockout tumor cells. In the SJSA-1 osteosarcoma model, its anti-tumor activity was obvious, and it could significantly promote tumor regression without obvious toxicity or side effects [54, 55]. In addition, AMG232 can be combined with other cytotoxic drugs to improve its anti-tumor activity without obvious side effects [56]. Based on these findings, researchers have carried out more in-depth research, but no other compounds have entered clinical trials [57–60].

AMG232, MEK inhibitors and DNA damage-induced chemotherapy have synergistic tumor-killing effects, and combination therapy can significantly increase anti-AML effects in vivo [61]. Compared with RG7112, the selectivity of AMG232 for p53 wild-type cells was higher than that for p53 mutant cells, and glioblastoma stem cells were highly sensitive to AMG232 [62]. In addition, the combined application of AMG232 and anti-PD-1 antibodies can enhance the killing effect of T cells on tumor cells and had the potential to overcome the drug resistance of immunotherapy [63]. In a phase I trial, AMG232 showed good safety and pharmacokinetic parameters in patients with p53 WT advanced solid tumor or multiple myeloma and could control the progression of the disease [64]. A phase I clinical trial on metastatic cutaneous melanoma demonstrated that AMG232 combined with trametinib or dabrafenib had a tolerated dose, safety and pharmacokinetic parameters, and early anti-tumor activity [65]. A Phase I trial on AML patients showed that although AMG232 had serious gastrointestinal adverse reactions at high doses, it had objective pharmacokinetic parameters, targeting and clinical activity [66].

## NVP-CGM097

**Fig. 13:**
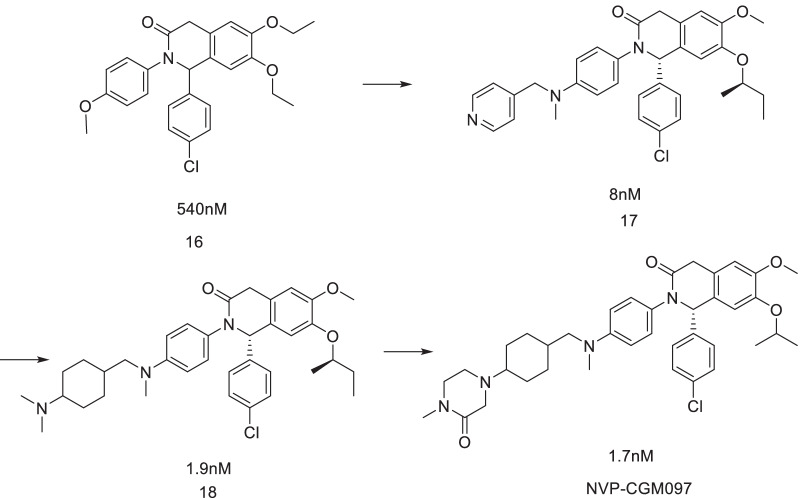
Discovery of NVP-CGM097

Dihydroisoquinolinone was selected as the scaffold of MDM2 inhibitors by virtual screening (Fig. 13). After preliminary structural optimization, Compound 17 was obtained, and its binding activity with MDM2 protein was 8 nM (TR-FRET). The anti-tumor activity in SJSA-1 cells was at the μM level. The cocrystal structure of Compound 17 with MDM2 protein showed that it had binding modes that were different from the existing MDM2 inhibitors. Butoxy and chlorobenzene occupied Leu26 and Trp23 cavities, respectively. Structure–activity relationship studies showed that these two parts had small structural modification potential. In the Phe19 cavity, groups can be introduced to enhance its binding to the cavity, and its 4-pyridine ring is surrounded by Tyr-67, Met-62 and Gln-72 amino acids of MDM2, which can significantly enhance its binding to MDM2 [67].

Through molecular docking, further studies on the binding of the Phe19 cavity showed that the substitution of the pyridine ring in the compound for naphthylamine or naphthyl compounds effectively enhanced its binding ability to the surrounding amino acids. For example, when the pyridine ring was substituted with piperidine and other rings, its affinity to MDM2 protein and cell activity were significantly enhanced, and at the same time, it showed good ADME parameters in vitro. Compound 18 was selected for PK experiments in rats. Compound 18 had typical PK parameters of tertiary amine compounds, with a wide distribution in vivo and long half-life. However, its in vivo clearance rate was low. The low oral bioavailability indicated that the absorption of the agent was not completely related to its in vitro solubility/permeability balance. It was also observed that its terminal dimethylamino group had a great influence on the activity. Based on this, the effects of different cyclic derivatives on their activities were further investigated. When this group was derivatized with imidazolidinone or piperazinone, the activity of the target compound was the strongest with good PK parameters and reduced in vivo distribution and half-life, and retained an intermediate in vivo clearance rate. Compared with compound 18, it had better absorption efficiency and oral bioavailability. Finally, NVP-CGM097 was obtained by ammonia methylation of piperazinone to enhance its permeability, which showed good cell activity, metabolic stability and PK parameters [68]. When comparing the binding ability of NVP-CGM097 and Nutlin-3a to MDM2 protein in different species, Nutlin-3a had little difference in affinity to MDM2 protein in different species, while NVP-CGM097 had great difference. The binding ability of NVP-CGM097 to human MDM2 was 16 times higher than that to dog MDM2, while the difference between Nutlin-3a and other species was larger. It is speculated that it may be due to the difference in the Leu-54 and Leu-57 amino acids. Therefore, it is important to study the stability of Nutlin-3a in monkey liver microsomes and the PK parameters in monkeys. Therefore, NVP-CGM097 was determined to be the most suitable clinical candidate.

**Fig. 14:**
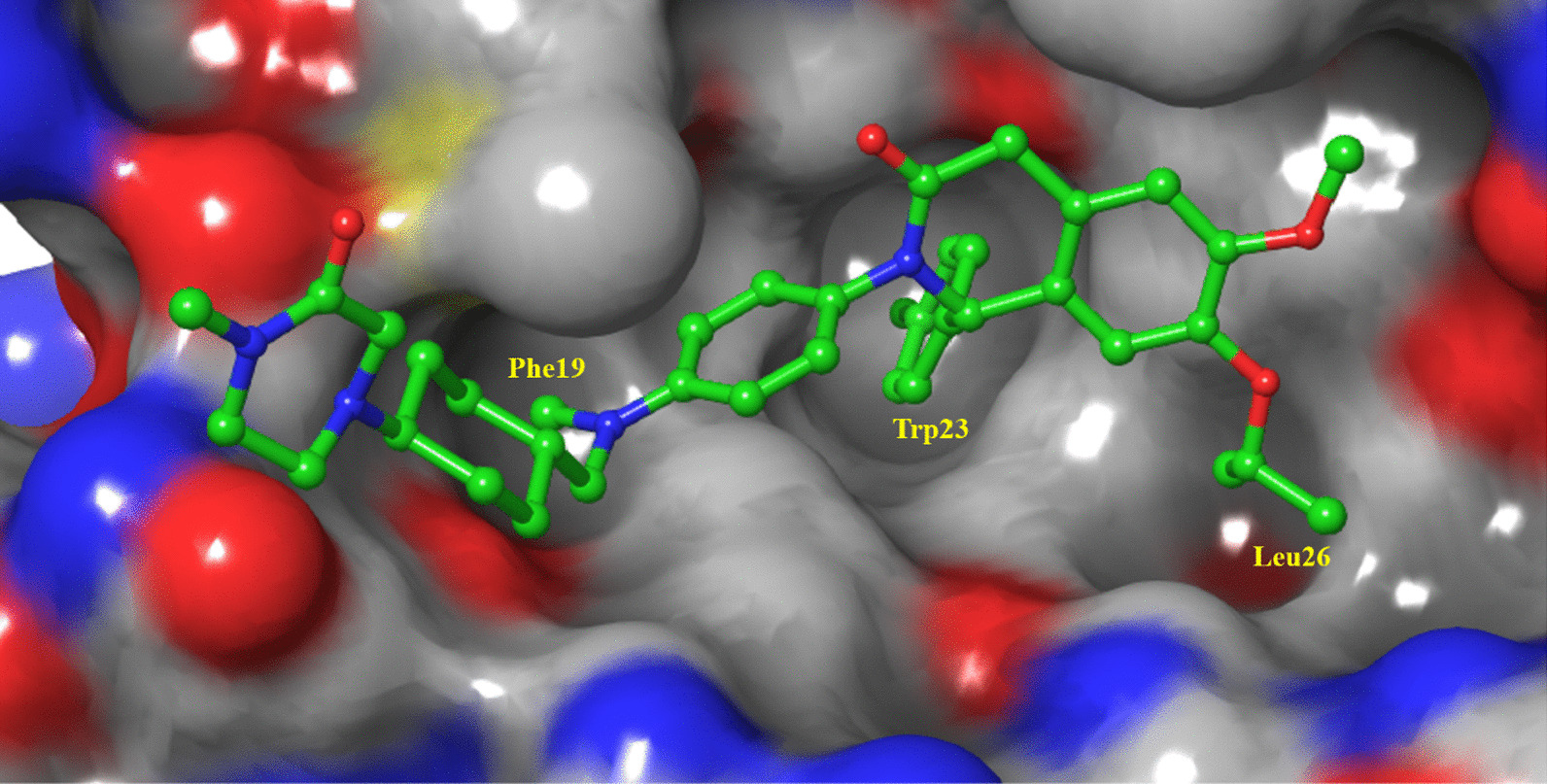
Binding pattern of NVP-CGM097 to MDM2 protein (PDB ID: 4ZYF)

The cocrystal structure of NVP-CGM097 and MDM2 protein is shown in Fig. 14, which showed that the dihydroisoquinolinone scaffold is located in the middle of the MDM2 active pocket as a scaffold to connect the three key groups to occupy the three key active cavities of Leu26, Trp23 and Phe19. The Leu-26 pocket was filled with isopropyl ether and methyl ether. The ether oxygen group could form water-mediated H-bond interaction with the hydroxyl of Tyr-100 and the carbonyl of Gln-24. The carbonyl group in the scaffold was also involved in the formation of H-bonds with the carbonyl group of Phe-55, which is helpful for the overall affinity of the molecule. More importantly, it induced the conformational constraints of its adjacent *N*-aryl side chain, so that it had a correct torsion angle to ideally enter the Phe19 active cavity ideally. *N*-Methyl piperazine can bind to the entrance of the Phe19 cavity in the water-rich region, perfectly located between the protein wall, and significantly enhance its affinity with the MDM2 protein [68].

NVP-CGM097 showed strong tumor inhibitory activity with derivative inhibitors in patients with B-cell acute lymphoblastic leukemia [69]. It had the effect of synergistic anti-BRAF (v-raf murine sarcoma viral oncogene homolog B1) mutation melanoma cell proliferation with RAF kinase inhibitor [70], and synergistic anti-ALK (anaplastic lymphoma kinase) mutation neuroblastoma with ALK inhibitor and prolonged survival [71]. The p53 mutant BON1 and NCI-H727 cells were resistant to NVP-CGM097, but when it was combined with 5-fluorouracil, it played a potential role in treating neuroendocrine tumors by increasing the expression of p53 and p21 [72]. NVP-CGM097 can also reverse the multidrug resistance of tumor caused by ABCB1 (ATP binding cassette subfamily B member 1) by blocking the drug efflux mediated by ABCB1 and improving the therapeutic effect of chemotherapy [73]. A phase I clinical trial demonstrated that NVP-CGM097 can control solid tumor progression by activating the p53 pathway and has acceptable pharmacokinetic parameters and safety [74].

## HDM201

HDM201 was also designed by Novartis. The design inspiration was mainly derived from the conformational controversy of MDM2 protein binding to dihydroisoquinolinone compounds. Although the specific structural modification process of HDM201 has not been fully published, its research ideas have been reported in the literature. Therefore, the research ideas and the current research process are summarized to provide inspiration for the design of other types of MDM2 inhibitors.

**Fig. 15:**
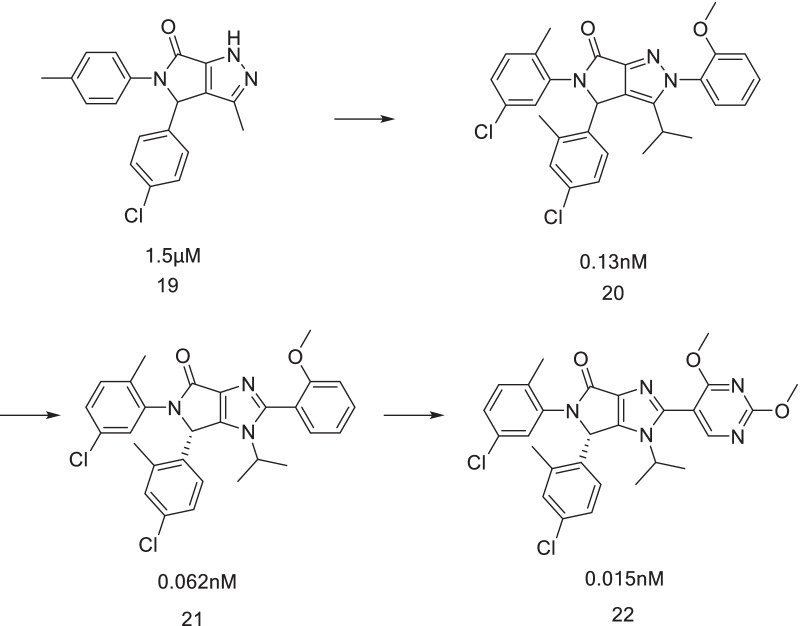
Discovery of HDM201

When NVP-CGM097 binds to the MDM2 protein, the scaffold of dihydroisoquinolinone is not completely planar, and therefore, the combination of NVP-CGM097 and the MDM2 protein results in a conformational energy loss, which can be overcome by van der Waals forces or hydrogen bonding interactions with binding sites. However, it is reasonable to believe that the planar scaffold is beneficial for the binding of the compound to MDM2. Therefore, it was assumed that the five-membered lactam ring replaces the six-membered lactam ring of the dihydroisoquinolinone nucleus (Fig. 15). When it was fused with the aromatic ring, it was completely planar and highlighted the substituent, and this idea was verified by molecular simulation. Subsequently, Compound 19 was obtained, and its binding capacity with MDM2 was 1.5 μM. Through simple structural optimization, two methyl groups were introduced into the benzene ring to increase the steric hindrance, so that the two benzene rings were rotated to a suitable angle. After structural analysis, there were still modifiable sites for introduction of phenyl at the N2 position, which led to the discovery of Compound 20 with further improved binding ability to 0.13 nM and cell activity to 90 nM [75].

Subsequently, the effects of a pyrazole ring, pyrrole ring and imidazole ring in the scaffold on the activity of the compound were compared. It was found that the imidazole ring reduced the lipophilicity of the compound, and therefore, an intermediate scaffold was prepared with pyrrolidone and imidazole rings, which resulted in Compound 21. Metasite analysis showed that the methoxy group embedded in the Phe19 cavity was easy to oxidize and metabolize, which led to the rapid metabolic clearance of the compound. Therefore, it was proposed to increase the appropriate substituent to reduce its electronic density. Finally, Compound 22 was obtained, which was effective in the SJSA-1 tumor model without affecting animal weight, compared with NVP-CGM097, which had more potential as the clinical candidate [76]. Subsequent structural optimization has not been reported, but this structure is very similar to HDM201.

HDM201 could selectively induce cell cycle arrest and apoptosis of p53 wild-type tumors and had dose-dependent PKPD parameters to inhibit SJSA-1 in vivo growth [77–79]. In contrast to NVP-CGM097, HDM201 intermittent pulse high-dose treatment can induce PUMA (p53 upregulated modulator of apoptosis) expression and apoptosis in the preclinical model to achieve in vivo anti-tumor activity [80]. The PKPD model of HDM201 also demonstrated that the anti-tumor activity of HDM201 was not scheduled dependent, but was related to cumulative dose [81], suggesting the feasibility of clinical intermittent administration to reduce the common hematological toxicity of MDM2 inhibitors. HDM201 combined with FLT3 (Fms-like tyrosine kinase 3) inhibitor can specifically induce apoptosis and death of FLT3-ITD positive TP53 wild-type AML cells [82], indicating that HDM201 has the potential to be combined with other targeted agents. Phase I clinical results showed that HDM201 had good pharmacokinetic parameters and safety, and there was no significant difference between its safety and tumor type and treatment plan, and it was prone to thrombocytopenia [83]. Clinical research on single-dose administration is ongoing. HDM201 showed promising antileukemia activity in patients with wild-type p53 [84]. The phase Ib trial with AML patients showed that AML patients had a good tolerance and therapeutic response to HDM201 combined with Bcl-2 inhibitors [85]. The safety and clinical efficacy of HDM201 against liposarcoma in combination with LEE011 (a CDK4/6 inhibitor) were also confirmed [86]. In AML patients with relapse after allogeneic stem cell transplantation, HDM201 also had good tolerance and clinical activity [87].

## Discussion

A study on the protein–protein interaction of p53–MDM2 showed that the expression of p53 protein could be regulated by MDM2. MDM2 can directly bind to the p53 protein, so that p53 protein would not be released and activated in time when stimulated, so that it could directly inactivate or weaken its transcriptional function. The unique RING domain of the MDM2 protein promotes the formation of dimers, which induces the transfer of the p53 protein from the nucleus to the cytoplasm, which abolishes its transcriptional function. At the same time, studies have shown that MDM2 can cooperate with E4 ligase to degrade p53. At present, nine small-molecule MDM2 inhibitors belonging to different structural types have entered clinical trials (Table 1). However, the clinical research data (such as for RG7112, RG7388 and AMG232) have shown that the progression of MDM2 inhibitors in clinical trials is hindered by various problems. For example, RG7112 was an outstanding candidate in preclinical research and demonstrated strong anti-tumor proliferation activity against various tumor cells. However, its efficacy in clinical research was not obvious, almost all tumor patients had no obvious tumor response, and nearly half of the patients had multiple serious adverse reactions (such as neutropenia and thrombocytopenia) [26]. A clinical study of RG7388 also showed similar phenomenon, with no obvious p53 activation and serious dose-dependent side effects [88]. In addition to the above problems, the mutation of ccfDNA (circulating cell-free DNA) was also found in the clinical study of AMG232. After continuous administration of medication, the mutation frequency gradually increased, and a variety of p53 mutations also occurred, resulting in severe drug resistance [65]. The main defects can be summarized as the following: (1) the clinical efficacy of MDM2 inhibitors is inconsistent with the results of in vitro studies, indicating that the underlying mechanism and a novel strategy need to be explored; (2) the hematological toxicity with activation of p53 in the bone marrow, which maybe solved by optimizing the dosing regimen (have been carried out by several clinical studies); (3) targeting MDM2 only has an inhibitory effect on p53 WT tumors, and however, p53 mutation can be found in more than 50% of cancers, and MDM2 inhibitors have been shown to induce p53 mutations in experimental systems.

**Table 1:** Lists of small-molecule MDM2 inhibitors in clinical trials

Drug	Disease	Combination	Phase	Status	Trial No
RG7112 (RO5045337)	Advanced solid tumors		I	Completed	NCT00559533
	Hematologic neoplasm		I	Completed	NCT00623870
	Solid tumors		I	Completed	NCT01164033
	Sarcoma	Doxorubicin	Ib	Completed	NCT01605526
	AML	Cytarabine	Ib	Completed	NCT01635296
	Sarcoma		I	Completed	NCT01143740
	CML, neoplasms, AML		I	Completed	NCT01677780
RG7388 (Idasanutlin)	Advanced malignancies, except leukemia		I	Completed	NCT01462175
	Solid tumors		I	Completed	NCT03362723
	AML	Idarubicin Daunorubicin Cytarabine	I/Ib	Completed	NCT01773408
	Relapsed and refractory AML	Cytarabine	III	Terminated	NCT02545283
	Non-Hodgkin’s lymphoma	Obinutuzumab Rituximab	I/Ib	Terminated	NCT02624986
	Relapsed and refractory AML	venetoclax	Ib	Completed	NCT02670044
	Relapsed and refractory follicular lymphoma, relapsed and refractory diffuse large B-cell lymphoma	Obinutuzumab Venetoclax Rituximab	Ib/II	Terminated	NCT03135262
	AML	Cytarabine Daunorubicin	Ib/II	Completed	NCT03850535
	Breast cancer	Atezolizumab	I/II	Terminated	NCT03566485
	Solid tumors		I	Completed	NCT02828930
	Polycythemia vera, essential thrombocythemia	Pegasys	I	Completed	NCT02407080
	Neoplasms	Posaconazole	I	Completed	NCT01901172
	AML, acute lymphocytic leukemia, neuroblastoma, solid tumors	Cyclophosphamide Topotecan Fludarabine Cytarabine	I/II	Recruiting	NCT04029688
	Relapsed multiple myeloma	Ixazomib Dexamethasone venetoclax	I/II	Active, not recruiting	NCT02633059
	Solid tumors	Entrectinib Alectinib Atezolizumab Ipatasertib Trastuzumab emtansine Inavolisib Belvarafenib Pralsetinib	II	Recruiting	NCT04589845
	Colorectal cancer	Regorafenib Atezolizumab Imprime PGG Bevacizumab Isatuximab Selicrelumab AB928 Genetic: LOAd703	I/II	Recruiting	NCT03555149
	Glioblastoma	APG101 Alectinib Atezolizumab Vismodegib Temsirolimus Palbociclib	I/II	Recruiting	NCT03158389
AMG232 (KRT-232)	Advanced solid tumors, multiple myeloma		I	Completed	NCT01723020
	AML	Trametinib	I	Completed	NCT02016729
	Metastatic melanoma	Trametinib Dabrafenib	Ib/IIa	Completed	NCT02110355
	AML, relapsed and refractory AML	Decitabine	I	Suspended	NCT03041688
	Soft tissue sarcoma	Radiation therapy	Ib	Recruiting	NCT03217266
	Polycythemia vera	Ruxolitinib	II	Active, not recruiting	NCT03669965
	Relapsed multiple myeloma	Carfilzomib Dexamethasone Lenalidomide	I	Recruiting	NCT03031730
	Brain cancer	Radiation therapy	I	Suspended	NCT03107780
	AML	Cytarabine Idarubicin HCI	Ib	Recruiting	NCT04190550
APG-115 (AA-115)	Advanced solid tumors, lymphomas		I	Completed	NCT02935907
	Metastatic melanomas, advanced solid tumors	Pembrolizumab	Ib/II	Recruiting	NCT03611868
	Salivary gland carcinoma	Carboplatin	I/II	Recruiting	NCT03781986
	AML, acute lymphocytic leukemia, neuroblastoma	Azacitidine Cytarabine	Ib	Recruiting	NCT04275518
	AML	5-Azacitidine	Ib/II	Recruiting	NCT04358393
	Liposarcoma, advanced solid tumors	Toripalimab	Ib/II	Recruiting	NCT04785196
	T-prolymphocytic leukemia	APG-2575	IIa	Recruiting	NCT04496349
CGM-097	Advanced solid tumors with TP53wt		I	Completed	NCT01760525
HDM201	Liposarcoma	Ribociclib	Ib/II	Completed	NCT02343172
	Uveal melanoma	LXS196	I	Completed	NCT02601378
	Advanced solid and hematological TP53wt tumors	Ancillary treatment	I	Completed	NCT02143635
	AML		I/II	Withdrawn	NCT03760445
	Advanced/metastatic colorectal cancer	Trametinib	I	Recruiting	NCT03714958
	Myelofibrosis	Ruxolitinib	I/II	Recruiting	NCT04097821
	Colorectal cancer, nonsmall cell lung carcinoma, triple negative breast cancer, renal cell carcinoma	Spartalizumab	I	Completed	NCT02890069
	Malignant solid tumors	Ribociclib	II	Recruiting	NCT04116541
	AML	Midostaurin	I	Recruiting	NCT04496999
	AML, myelodysplastic syndromes	MBG453	Ib	Recruiting	NCT03940352
DS-3032b (Milademetan)	Advanced solid tumors, lymphomas		I	Completed	NCT01877382
	Relapsed and refractory AML		I	Completed	NCT03671564
	AML	Quizartinib	I	Terminated	NCT03552029
	AML, myelodysplastic syndromes	5-Azacitidine	I	Terminated	NCT023199369
	AML, relapsed and refractory AML	Cytarabine Venetoclax	I/II	Completed	NCT03634228
	Myeloma		I	Terminated	NCT02579824
SAR405838	Neoplasm malignant	Pimasertib	I	Completed	NCT01985191
	Neoplasm malignant		I	Completed	NCT016636479
MK-8242	AML	Cytarabine	I	Terminated	NCT01451437
	Solid tumors		I	Terminated	NCT01463696

When the cellular DNA is damaged, stimulated by abnormal growth signals, and affected by chemical drugs or ultraviolet rays, the p53 gene is transcribed and translated into protein, and various kinases are activated to phosphorylate the p53 protein. The accumulated phosphorylated p53 protein in the nucleus promotes cell cycle arrest for self-repair. While the MDM2 is overexpressed, the function of p53 in protecting the cell will be disrupted. Therefore, the clinical efficacy of MDM2 inhibitors may mainly rely on the reversible cell cycle arrest rather than direct induction of cell death through p53-dependent signaling pathway [89–91]. Taking Nutlin-3a as an example, the sensitivity of MDM2 inhibitors against cancer cells was influenced by more criteria other than p53 mutation [92]. The wild-type p53-PUMA pathway was found to have the potential to drive the metabolic switch of cancer cells, and wild-type p53 is required for the maintenance of cancer cell growth and glycolysis in several cancers [93], indicating that activation of p53 in some cases may have the opposite effect, which was consistent with results from other studies [94–96]. MDM2 inhibitors may promote the emergence of p53 mutations and lead to genomic instability through a p53-independent mechanism, resulting in acquired resistance against MDM2 inhibitors [97–99]. In addition, although missense mutations in MDM2 are rare, multiple isoforms of MDM2 protein generated by alternative promoters and alternative proteins have been observed, and the change in the sequence may disrupt the N-terminal p53-binding domain to reduce the efficacy of MDM2 inhibitors and promote tumor progression [100–103]. All of the above factors may be the reasons for the unsatisfactory clinical effects and development of resistance to MDM2 inhibitors. Although the biological classifier based on the genome-wide association has the potential to discriminate response to MDM2-inhibitor therapy [104], novel treatment strategies based on MDM2 need to be explored.

Studies have shown that although MDMX has no obvious regulatory effect on the expression of p53 protein, it can bind to MDM2 protein to form dimers, improve the activity of its E3 ubiquitin ligase and form the above RING domain, thereby further inactivating the p53 protein, which showed considerable efficacy with MDM2. Inhibition of MDM2 alone cannot completely release and activate p53 protein [25, 105], which can induce the overexpression of MDMX, thereby reducing the effect of MDM2 inhibitors and promoting drug resistance [106]. Meanwhile, in some tumors, such as liver cancer and breast cancer, the inactivation of p53 protein is mainly related to the overexpression of MDMX, and therefore, MDM2 inhibitors have shown little effect on such tumors [107]. Studies have shown that the application of MDM2/MDMX dual-target inhibitors can activate higher levels of p53 protein than single-target inhibitors with a better anticancer efficacy, indicating that the simultaneous inhibition of MDM2 and MDMX proteins can effectively regulate the expression of p53 protein. It seems that the development of MDM2/MDMX dual-target inhibitors may solve the problems existing in the clinical research of the above MDM2 inhibitors and has great practical significance for the treatment of related tumors [97, 108, 109].

However, at present, the development of MDM2/MDMX dual-target inhibitors is slow, although the interaction between p53 and MDM2/MDMX occurs through the same key amino acid residues (Phe19, Trp23 and Leu26), the binding cavity of MDMX is more flexible than that of MDM2, which makes the existing MDM2 inhibitors with rigid scaffolds unable to conform to the conformational flexibility in the active cavity of the MDMX protein. However, the MDMX inhibitor SJ-172550 was reported to be extremely unstable [110]. It easily deteriorates in solution, and the basis of its specific role is not clear. Only indole-acetylureas and pseudopeptides have been reported to have dual protein inhibitory effects onMDM2 and MDMX [111–113], with few structural types, unclear mechanisms of action, and no further studies.

In addition to small-molecule inhibitors, another promising class of MDM2/MDMX inhibitors is cell-penetrating stapled α-helical peptides. The most promising is ATSP-7041 and its analog ALRN-6924 [114, 115]. ALRN-6924 significantly improved survival in a xenograft model of AML [116]. Clinically, ALRN-6924 is being evaluated as a monotherapy and in combination with cytarabine in patients with hematological malignancies (NCT02909972) and has entered a Phase I/II clinical trial in patients with advanced solid tumors or with preserved wild-type p53 lymphoma (NCT02264613). ALRN-6924 was well tolerated, and the most common adverse side effect was gastrointestinal [115].

The dual-target inhibitor based on MDM2 is a promising way to solve the defects of MDM2 inhibitors. The MDM2/XIAP (X-linked inhibitor of apoptosis protein) dual-target inhibitor MX69 can simultaneously inhibit XIAP and activate p53 to exert anti-tumor activity. Importantly, it showed little effect on normal human hematopoietic function in vitro and was well tolerated in animal models [117], indicating that it had the potential to avoid adverse reactions in hematopoietic system of MDM2 inhibitors. He et al. discovered an oral dual inhibitor of MDM2 and HDAC (histone deacetylase), which had excellent anti-tumor effect in xenograft models [118]. Targeting MDM2/TPSO (translocator protein) [119], MDM2/PKC (protein kinase C) [120], MDM2/NF-kB (nuclear factor-kappa B) [121], MDM2/Bcl-2 (B-cell lymphoma-2) [122] and MDM2/NFAT1 (nuclear factor of activated T-cells 1) [123] had good anti-tumor activity and had the potential to reduce the adverse reactions of MDM2 inhibitors. Targeted MDM2 degradation also had the potential to reduce the dose-dependent hematological toxicity of MDM2 inhibitors [124–128]. In addition, MDM2 inhibitors have the potential to be incorporated into cyclotherapy, since low-dose MDM2 inhibitors could arrest the cell cycle and prevent S-Phase and M-Phase drug damage to p53 proficient normal cells. However, no multitarget inhibitors or degradation agents or cyclotherapies related to MDM2 have entered the clinical trials, suggesting that the related structures need to be further optimized to improve their physicochemical properties and promote their clinical development.

## Conclusion

In summary, the design and development of anti-tumor drugs with new mechanisms targeting MDM2–p53 are one of the hotspots in the field of global tumor drug research and development. However, the specificity of MDM2–p53 protein interaction brings considerable difficulties to the development of small-molecule inhibitors, resulting in only a handful candidates under clinical trials, and no marketed drugs targeting MDM2. The multi-target strategy or targeted degradation strategy based on MDM2 has the potential to improve the clinical defects of MDM2 inhibitors, but more evidence is still needed to confirm its clinical application value.

## Data Availability

Not applicable.

## Abbreviations

ABCB1: ATP binding cassette subfamily B member 1
ALK: Anaplastic lymphoma kinase
AML: Acute myeloid leukemia
Bcl-2: B-cell lymphoma-2
BRAF: V-raf murine sarcoma viral oncogene homolog B1
CML: Chronic myeloid leukemia
CPM: China Pharmaceutical Pipeline Monitor database
FLT3: Fms-like tyrosine kinase 3
HDAC: Histone deacetylase
HIF1α: Hypoxia inducible factor-1α
Keap1: Kelch-like ECH-associated protein 1
MAPK: Mitogen-activated protein kinase
MDM2: Mouse double minute 2
MDMX: Mouse double minute X
MEK: MAP kinase kinase
NFAT1: Nuclear factor of activated T-cells 1
NF-kB: Nuclear factor-kappa B
Nrf2: Nuclear factor E2-related factor 2
PKC: Protein kinase C
PPI: Protein–protein interaction
PUMA: P53 upregulated modulator of apoptosis
TPSO: Translocator protein
UBE4B: Ubiquitination factor E4B
VHL: Von Hippel–Lindau
XIAP: X-linked inhibitor of apoptosis protein
